# Effects of a *Lacticaseibacillus* Mix on Behavioural, Biochemical, and Gut Microbial Outcomes of Male Mice following Chronic Restraint Stress

**DOI:** 10.3390/nu15214635

**Published:** 2023-10-31

**Authors:** Vivien Letenneur, Magali Monnoye, Catherine Philippe, Sophie Holowacz, Sylvie Rabot, Patricia Lepage, Elsa Jacouton, Laurent Naudon

**Affiliations:** 1 Université Paris-Saclay, INRAE, AgroParisTech, Micalis Institute, 78350 Jouy-en-Josas, France; vivien.letenneur@gmail.com (V.L.); magali.monnoye@inrae.fr (M.M.); catherine.philippe14@wanadoo.fr (C.P.); sylvie.rabot@inrae.fr (S.R.); patricia.lepage@inrae.fr (P.L.); 2 PiLeJe Laboratoire, Carré Suffren, 31–35 Rue de la Fédération, CEDEX 15, 75015 Paris, France; s.holowacz@pileje.com (S.H.); e.jacouton@pileje.com (E.J.); 3 Université Paris-Saclay, INRAE, AgroParisTech, CNRS, Micalis Institute, 78350 Jouy-en-Josas, France

**Keywords:** psychobiotics, microbiota–gut–brain-axis, chronic stress, anxiety behaviour

## Abstract

The effect of supplementation with *Lactobacillus* strains to prevent the consequences of chronic stress on anxiety in mouse strains sensitive to stress and the consequences on gut microbiota have been relatively unexplored. Thus, we administered a *Lacticaseibacillus casei* LA205 and *Lacticaseibacillus paracasei* LA903 mix to male BALB/cByJrj mice two weeks before and during 21-day chronic restraint stress (CRS) (non-stressed/solvent (NS-PBS), non-stressed/probiotics (NS-Probio), CRS/solvent (S-PBS), CRS/probiotics (S-Probio)). CRS resulted in lower body weight and coat state alteration, which were attenuated by the probiotic mix. S-Probio mice showed less stress-associated anxiety-like behaviours than their NS counterpart, while no difference was seen in PBS mice. Serum corticosterone levels were significantly higher in the S-Probio group than in other groups. In the hippocampus, mRNA expression of dopamine and serotonin transporters was lower in S-Probio than in S-PBS mice. Few differences in bacterial genera proportions were detected, with a lower relative abundance of *Alistipes* in S-Probio vs. S-PBS. CRS was accompanied by a decrease in the proportion of caecal acetate in S-PBS mice vs. NS-PBS, but not in the intervention groups. These data show that the probiotic mix could contribute to better coping with chronic stress, although the precise bacterial mechanism is still under investigation.

## 1. Introduction

In 2019, the World Health Organization reported that there were 970 million people (1 in 8) living with mental disorders, including anxiety and depression. In recent years, mental disorders have increased significantly worldwide, in part due to the Coronavirus Disease 2019 pandemic and other circumstances leading to exposure to chronic stress [1,2]. However, we are not equal in our response to stress, with some people being more or less likely to suffer from mood disorders. In recent years, several studies have focused on the capacity for resilience and the mechanisms to cope with stressors, including oxidative stress, neuronal plasticity, and blood–brain barrier permeability [3,4]. Psychological and mental well-being is an integral part of health, which has led to the search for new preventive and therapeutic approaches complementing standard psychological care and pharmacological treatments. In this regard, psychobiotics have been proposed as a novel class of probiotics with beneficial effects on the health of patients with mental disorders [5].

During the last few decades, gnotobiotic animals and antibiotic interventions have provided evidence that the gut microbiota influences stress-related behaviours [6,7,8]. The concept of the microbiota–gut–brain axis (GBA), referring to the bidirectional communication pathways between the gut and the brain, has emerged. These communication pathways include endocrine (cortisol), immune (cytokines), neural (vagus nerve and enteric nervous system), and metabolic (short chain fatty acid (SCFA)) pathways [9]. In recent years, mouse models of anxiety and depression-like behaviours induced by chronic stress have been used to decipher GBA interactions [10,11,12]. Some of these studies revealed that stress-induced microbiota alterations (characterized in part by a depletion in the genus *Lactobacillus*) were sufficient to produce behavioural impairment. Since then, growing evidence has shown that supplementation with probiotic strains of *Lactobacillus* confers resilience to anxiety and or depressive behaviours by modulating inflammation, corticosterone, neurotransmitters such as serotonin (5-HT), and the microbiota [13,14,15,16,17,18,19]. In addition, a few studies have reported beneficial effects of probiotics in students undergoing the stress of an academic examination or in adults suffering from chronic stress [20,21,22,23,24,25], although recent meta-analyses have suggested an effect of probiotics on more sensitive individuals (suffering from depressive symptoms) than on healthy subjects [26,27]. Despite these promising studies, the mechanisms involved are far from being fully elucidated, particularly the mechanisms responsible for resilience or susceptibility to stressors. This is further complicated by the fact that the behavioural response and molecular pathways involved are strain-dependent, and there is considerable heterogeneity in the type and duration of stress applied and mouse lines used in the studies.

In the present study, supplementation with two probiotic strains, *Lacticaseibacillus casei* (*L. casei*) LA205 and *Lacticaseibacillus paracasei* (*L. paracasei*) LA903, was evaluated in the BALB/cByJrj murine model of chronic restraint stress (CRS). The LA205 and LA903 strains were chosen because their anxiolytic properties had previously been evaluated in an acute stress model with favourable results (PiLeJe data on file). BALB/cByJrj mice exhibit spontaneously elevated anxiety [28], making them good candidates to mimic the greater susceptibility to chronic stressors observed in some individuals. The preclinical model of CRS is widely used to mimic environmental stress in adults with good validity [29]. Using this model, we investigated the ability of the probiotic mix (PM) to help mice cope better with acute stress and counteract the CRS-induced cerebral and intestinal alterations.

## 2. Material and Methods

### 2.1. Animals

A total of 56 BALB/cByJrj male mice (Janvier Labs, Le Genest Saint Isle, France), aged 5 weeks on arrival, were housed, 3–4 mice per cage, under standard conditions (12:12 h light–dark cycle, lights on at 7:30 a.m., 22 °C). All animals had free access to food (standard diet (R03-40; SAFE)) and water. Each cage contained a transparent polycarbonate tunnel, a Nestlet for nest building (Serlab^®^, Montataire, France), wooden chew sticks (Safe^®^, Augy, France), and a sheet of absorbent paper towel. The animals were identified using subcutaneous transponders. On their arrival, mice were weighed to be allocated to four groups of similar weight (n = 14), named, respectively, NS-PBS, S-PBS, NS-Probio, and S-Probio, depending on whether they were stressed or not (S or NS) and whether they had received PM or not (Probio or PBS). Then, mice were weighed every week. Experimental procedures were carried out in accordance with EU Directive 2010/63/EU for animal experiments. They were approved by the Ethics Committee of the INRAe Research Center at Jouy-en-Josas (Comethea C2EA-45) and authorized by the French Research Ministry (APAFiS #26828).

### 2.2. Gavage Procedure

**Figure 1:**
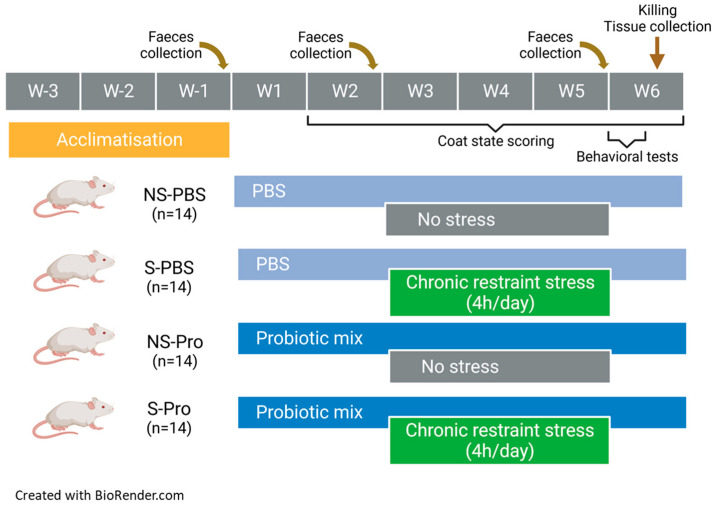
Study design.

By the third week of habituation after their arrival, mice were accustomed to the manual restraint and gavage procedure. During simulated gavage, no substance was administered. The gavage was initiated after the habituation period from week 1 to the 2 first days of week 6 (Figure 1). It was carried out each day at the beginning of the morning. The NS-PBS and S-PBS mice received 200 µL of 1X phosphate-buffered saline (PBS) per gavage. The NS-Probio and S-Probio mice received 200 µL of a probiotic mix (2 × 10^9^ CFU) of *Lactobacillus casei* LA205 and *Lactobacillus paracasei* LA903 in 1X PBS.

### 2.3. Chronic Restraint Stress Procedure

CRS of a duration of 4 h/day was initiated on the 3rd week of gavage for a duration of 3 weeks (Figure 1). CRS was performed in the morning after gavage. The restraint tubes were made from 50 mL Falcon© tubes pierced at both ends to allow air and tail passage.

### 2.4. Faecal Pellet Collection

Fresh pellets were collected in the morning on the day before the first gavage (T1), the first day of CRS (T2), and the day of behavioural (T3) tests. Each mouse was placed in a plastic jar; 5–6 pellets were collected, placed in tubes, and then quickly frozen and stored at −80 °C for later analysis.

### 2.5. Coat State

Once a week, the coat state of mice was assessed in eight different body parts: head, neck, dorsal coat, ventral coat, tail, forepaws, hind paws, and genital region. A score of 0 for a coat in a good state or a score of 1 for a dirty coat was given for each of these areas. Total score was obtained from the sum of the scores of each body part [30].

### 2.6. Behavioural Tests

All behavioural tests assessed anxiety. They were carried out over the two days following the last day of the CRS (Figure 1) in a dedicated room in the early afternoon (1–4 pm) with random selection of the order of the groups. Each session was recorded by a video camera placed above the behavioural apparatus. The videos were processed offline using video tracking software, ANY-maze 7.2 software (Stoelting Co., Dublin, Ireland). Some parameters were nevertheless evaluated directly by the manipulators present in the room but standing back from the device, such as the rearing and the evaluation of the number of faecal pellets at the end of each session.

### 2.7. Open Field Test

On the first day of testing, the test was conducted in a square open field (OF) of 45 × 45 cm and a 25 cm height with opaque walls. The mouse was placed in the bottom right corner of the OF and filmed for 6 min by a camera placed above the OF. To define the zones, the surface of the OF was divided into 25 equal squares, and the 9 central squares constituted the central zone. The “corner” zones were made up of one square. The number of visits and time spent in each of the zones (periphery, centre, and corners) were recorded, as well as the number of rearing, grooming, and faeces at the end of the session. If a mouse did not visit at least two corners and/or travelled a total of less than 2 m during the test, it was excluded from the analysis (adapted from [31]).

### 2.8. Elevated plus Maze Test

On the second day of testing, the test apparatus was a grey maze in the shape of a cross composed of four arms (30 cm long, 5 cm wide). Two opposite arms were surrounded on three faces by 16 cm high walls (closed arms), while the other two opposite arms were open (open arms). The maze was raised to a height of 50 cm from the floor and was slightly illuminated from the top (about 50 lux). At the beginning of the test, the mouse was placed in the intersection square, facing an open arm, which is considered an aversive area. Number of visits and time spent in the open and closed arms were recorded for 5 min. The results were expressed as the percentage of the number of visits (or time spent) in the open arms over the overall number of visits (or time spent) in both closed and open arms. When the total number of arms visited was less than 3, the mouse was excluded from the analysis. Other parameters measured were the number of visits and time spent in the centre and at the ends of open arms, number of head bendings, and number of faecal pellets at the end of the session [32].

### 2.9. Euthanasia and Blood and Tissue Collection

The day after elevated plus maze test, mice were euthanized via decapitation (Figure 1). The trunkal blood was collected in a plastic tube (MiniCollect^®^ 0.5/0.8 mL CAT Serum Sep Clot Activator, Greiner Bio-One, Madrid, Spain). After 3 h of clotting at room temperature, the samples were centrifuged at 2000× *g* for 10 min. The supernatant serum was collected and stored in aliquots at −80 °C. After sacrifice, spleen and adrenal glands were collected and weighed. The intestinal tract was removed and dissected on ice; small intestine and colon were gently flushed with PBS (pH 7.4) to remove residual digestive contents, and 1 cm long tissue fragments were excised from the ileum and the upper part of colon and transferred into cryotubes filled with Ambion^®^ RNA later Stabilization Solution (Invitrogen, Carlsbad, CA, USA). After 24 h at room temperature, the cryotubes containing intestinal tissues were stored at −80 °C. The caecum was dissected; it was weighed full and then empty. The caecal contents were frozen on dry ice and then stored at −80 °C. Brains were quickly removed from the craniums. The hypothalamus, hippocampus, and prefrontal cortex were dissected out on ice and transferred into cryotubes filled with Ambion^®^ RNA later for 24 h at room temperature, then stored at −80 °C.

### 2.10. Microbiota Analysis

#### 2.10.1. Analysis of Faecal Microbiota Composition

DNA was extracted from faecal samples using the QIAamp^®^ PowerFecal^®^ Pro DNA Kit (Qiagen, Hilden, Germany) following the provided protocol. Concentration and purity of DNA were checked using spectrophotometry (Nanodrop, Fisher Scientific, Strasbourg, France). The DNA was diluted to obtain a concentration of approximately 50 ng/µL, then amplified via PCR using the Phanta Max Super-Fidelity Kit (Vazyme, Nanjing, China) and 16S V3-V4 region primers (forward primer: 5′ ACG GRA GGC AGC AG 3′; reverse primer: 5′ TACCAGGGTATCTAATCCT 3′). Amplicon size was checked on 1% agarose gel in TAE 1X with Midori Green Advance (Nippon Genetics Europe, Düren, Germany) and 1 kb ladder (New England Biolabs, Evry-Courcouronnes, France). The expected amplicon length was 457 pb. Amplicons were transferred on MicroAmp™ Optical 96-Well Reaction Plate (Applied Biosystems, Fisher Scientific) and sent for sequencing library construction and Illumina sequencing to the GenoToul GeT-Biopuces platform (Toulouse, France). Sequences were analysed using R combining dada2 v.1.26 [33] and FROGS 4.0.0 [34] workflows. Adapters were removed (cutadapt v. 3.5), and forward and reverse reads were filtered (dada2 filterAndTrim function; truncation length: 200 bp). The error model was then calculated using the learnErrors function. Then, the dada2 core sample inference algorithm was executed. Forward and reverse reads were merged with a minimum overlap of 20 bp. Chimeras were detected and removed using the vsearch tool according to FROGS v4.0.0 guidelines. Reads were grouped into ASVs with dada2. ASVs with global abundance lower than 0.005% were removed from the following analysis with FROGS filters. The ASVs in the sequence table were then assigned to bacterial taxa using FROGS affiliation with the Silva 138 database [33]. 16S rRNA amplicon sequencing biostatistical analyses and graphs were then performed in Rstudio (v4.2.2) with package Phyloseq (v1.42.0), Phyloseq.extended (v0.1.1.9), ade4, and ggplot2.

#### 2.10.2. Short Chain Fatty Acid (SCFAs) Analysis

Cecal contents were water-extracted, and proteins were precipitated with phosphotungstic acid. A total of 0.3 µL supernatant was analysed for SCFA on a gas chromatograph (Agilent 7890B, Agilent Technologies, Les Ulis, France) equipped with a split–splitless injector, a flame-ionisation detector, and a capillary column impregnated with SP 1000 (FSCAP Nukol, 15 m × 0.53 mm × 0.5 µm) (Supelco, Sigma-Aldrich, Saint-Quentin-Fallavier, France). The carrier gas (H2) flow rate was 10 mL/min, and the temperature curve was 100 °C for 10 min, followed by an increase from 100 to 180 °C at a rate of 20 °C/min and 2 min hold. The detector temperature was 240 °C. 2-ethylbutyrate (Supelco, Sigma Aldrich) was used as internal standard [35]. Samples were analysed in duplicate. Data were collected, and the peaks obtained were integrated using OpenLAB Chemstation software 2.3.53 (Agilent Technologies). Measures of acetate, propionate, butyrate, and branched and long chain fatty acids (isoSCFAs + valerate + caproate) were expressed in % of total SCFAs for statistical analysis.

### 2.11. Serum ELISA Assays

Serum concentrations of corticosterone, norepinephrine, kynurenine, tryptophan, and 5-HT were measured using specific ELISA kits (for corticosterone, Invitrogen^®^, Waltham, MA, USA; for the others, Immusmol^®^, Bordeaux, France) following the provided protocol. Samples were analysed in duplicate, optical density (OD) was measured using a multi-detection microplate reader (Infinite m200; Tecan, Männedorf, Switzerland), and concentration was extrapolated from the standard curve.

### 2.12. RT-qPCR for Intestinal and Brain Tissue

#### 2.12.1. RNA Extraction

Cerebral tissue was ground in lysis buffer (QIAzol^®^ Lysis Reagent) using a Potter-Elvehjem PTFE grinder compatible with 1.5 mL Eppendorf tubes. Intestinal tissues were ground under the same conditions with the exception of the use of an ultra turrax^®^ grinder (T25 basic IKA Labortechnik). Total RNA extraction was performed using RNeasy Plus mini kits (Qiagen). First, samples were put in 600 µL of lysis buffer (573 µL RLT Plus buffer (Qiagen), 4 µL dithiothreitol 1 M (Sigma-Aldrich), and 3 µL reagent DX (Qiagen)) with a stainless-steel bead (Qiagen) and homogenized at 1800 rpm for 2 min in a grinder (Powteq GT300, Grosseron, Couëron, France) in racks that had been previously frozen at −80 °C. Samples were centrifuged at 5000× *g* for 3 min at room temperature. The supernatants were transferred to new cryotubes and then frozen at −80 °C or used directly for extraction according to the provided protocol. No more than the equivalent volume of 20 mg of tissue was used to avoid clogging of columns. Quality of RNA eluates was checked on a 1% agarose gel with 6× GelRed^®^ Prestain Buffer with Blue Tracking Dyes (Biotium, Fremont, CA, USA), and RNA concentration and purity were checked on nanodrop (Fischer Scientific). RNA eluates were frozen at −80 °C until further use.

#### 2.12.2. Reverse Transcription

Reverse transcription of 1.5 µg of the RNA samples was performed using the High-Capacity cDNA reverse transcription kit (Applied Biosystems, Fisher Scientific), and the cDNA solution obtained was diluted (1:7 for intestine and 1:5 for brain) and stored at −20 °C.

#### 2.12.3. qPCR Brain Tissue

Quantitative PCR (qPCR) was performed on 1:5 diluted cDNA samples using Taqman Mastermix and TaqMan™ Gene Expression Assay (FAM or VIC) (detailed in Table 1) in MicroAmp™ Optical 96-Well Reaction Plate and analysed using StepOne™ Real-Time PCR System (Applied Biosystems, Fisher Scientific) using the following program: 2 min at 50 °C; 10 min at 95 °C, followed by 40 cycles of 15 s at 95 °C and 1 min at 60 °C. Ct were normalized by subtracting cycle threshold (CT) of the housekeeping gene (GAPDH or β-actin), and 2eΔΔCT was calculated (using the average of all animals for normalization as there was no “control group”) and used for statistical analysis.

#### 2.12.4. qPCR Intestine Tissue

Quantitative PCR (qPCR) was performed on 1:7 diluted cDNA samples using SYBR™ Green PCR Mastermix. Probes were designed by our team and produced by Eurofins Genomics (detailed in Table 2). All probes were designed to have a TM equal to 59 °C to standardize the qPCR. SYBR Green gene Expression Assay was made in MicroAmp™ Optical 96-Well Reaction Plate and analysed using StepOne™ Real-Time PCR System (Applied Biosystems, Fisher Scientific) using the following program: 10 min at 95 °C followed by 40 cycles of 15 s at 95 °C and 1 min at 59 °C. Ct were normalized by subtracting cycle threshold (CT) of the housekeeping gene (β-actin), and 2eΔΔCT was calculated (using the average of all animals for normalization as there was no “control group”) and used for statistical analysis.

### 2.13. Statistical Analysis

Systematically, a normality test (Shapiro-Wilk test) and a variance equality test (Fischer test) were performed on the data. In case of normal distribution and variance equality, data were expressed as mean ± SEM values obtained from the indicated number of mice, and we used one-way ANOVA, followed by Tukey’s multiple comparison test. In case of lack of normal distribution or of variance equality, data were expressed as median (interquartile range) and analysed using Kruskal–Wallis test, followed by Dunn’s multiple comparison test, to compare the three groups of mice. The level chosen for statistical significance was 5%. All calculations were performed using GraphPad Prism software (version 7.03, La Jolla, CA, USA).

Mann–Whitney test was used to compare the two groups, and Tukey HSD test was used to compare multiple groups. Differences between overall microbial communities were assessed on the PCA analyses by applying a Monte Carlo permutation test on the Between-Class Analysis (bca).

## 3. Results

### 3.1. CRS Led to a Decrease in Weight Gain, Which Was Significantly Attenuated in Probiotic-Supplemented Mice

**Figure 2:**
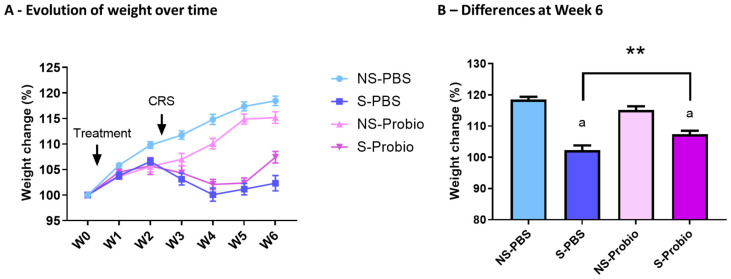
CRS led to a decrease in weight gain over time which was significantly attenuated in S-Probio mice compared to S-PBS mice. Evolution of weight in the 4 groups of mice (**A**) over time and (**B**) at Week 6. Two-way Anova analysis; results are expressed as mean ± SEM, n = 14. a *p* < 0.001 in S vs. NS within each treatment; ** *p* < 0.01 in S-PBS vs. S-Probio. CRS: chronic restrain stress; Treatment: PBS or probiotic mix; W: week; NS: non-stressed; S: stressed.

To investigate the effect of PM in the stress-induced model, we exposed mice to CRS for 3 weeks. PM administration was started before CRS and lasted until the end of the experiment (Figure 1). The weight of the mice was measured over 6 weeks. A two-way ANOVA analysis revealed a time effect (*p* < 0.001), a treatment effect (*p* < 0.001), as well as an interaction of both (*p* < 0.001) (Figure 2A). At week 6, S-Probio and S-PBS mice exhibited a significant stress-induced decrease in weight (Tukey’s multiple comparisons test, *p* < 0.001). However, this decrease was significantly lower in S-Probio mice than in S-PBS mice (*p* < 0.01) (Figure 2B). On the day of killing, a Dunn’s multiple comparison test following a Kruskal–Wallis test (*p* < 0.001) indicated that the average weight of S-PBS mice was significantly lower than that of NS-PBS mice, 27.4 ± 2.6 g (n = 14) vs. 29.7 ± 2.1 g (n = 14) (*p* < 0.001), whereas this was not the case in the probiotic groups (NS-Probio, mean, 28.8 ± 2.4 g (n = 14) vs. S-Probio, 27.6 ± 1.18 g (n = 14)).

### 3.2. CRS Induced a Deterioration in the Coat State over Time, Which Was Significantly Attenuated in Probiotic-Supplemented Mice

**Figure 3:**
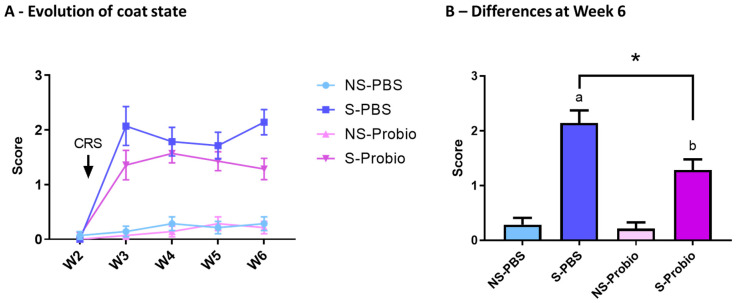
CRS induced a deterioration of coat state over time, which was significantly attenuated in S-Probio mice compared to S-PBS mice. Coat state evolution in the 4 groups of mice (**A**) over time (**B**) at Week 6. Two-way Anova analysis; results are expressed as mean ± SEM, n = 14. a *p* < 0.001, b *p* < 0.01 in S vs. NS; * *p* < 0.05 in S-PBS vs. S-Probio. CRS: chronic restrain stress; W: week; NS: non-stressed; S: stressed.

The coat state was assessed over time to estimate the level of anxiety in response to CRS and was significantly affected by time (*p* < 0.001), treatment (*p* < 0.001), and the interaction of both (*p* < 0.001) (two-way ANOVA) (Figure 3A). In particular, a post hoc test at week 6 indicated an increased score of coat deterioration in both S-PBS vs. NS-PBS and S-Probio vs. NS-Probio mice (*p* < 0.001 and *p* < 0.01, respectively, Tukey’s comparisons), reflecting greater anxiety. This deterioration was significantly weaker in S-Probio mice than in S-PBS mice (*p* < 0.05) (Figure 3B).

### 3.3. Probiotic-Supplemented CRS Mice Exhibited a Better Response to Additional Stress in Behavioural Tests

**Figure 4:**
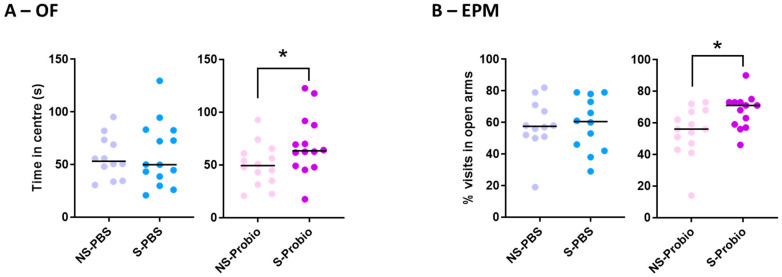
S-Probio mice (**A**) spent more time in the central area in the open field (OF) test and (**B**) made more visits in the open arms in the elevated plus maze (EPM) test than their NS counterparts. Mann-Whitney test; results are expressed as median, n = 12–14. * *p* < 0.05 in S vs. NS. NS: non-stressed; S: stressed.

The results obtained in the open-field (OF) and elevated plus maze (EPM) tests were compared using Kruskall–Wallis tests and did not differ between the four groups (Supplementary Table S1). This lack of difference, particularly for the key behaviours in these two anxiety tests (number and duration of visits to the centre in the OF and percentage of visits or time spent in the open arms in the EPM), indicated that CRS did not induce anxiety behaviour in either solvent- or probiotic-treated mice. However, when the effects of PM or PBS supplementation were analysed with the Mann–Whitney test, S-Probio mice spent more time in the central area of the OF and made more visits to the open arms of the EPM than their NS counterparts (Figure 4A,B).

### 3.4. PM Supplementation Induced Changes in Systemic Tryptophan Pathway and Hypothalamic–Pituitary–Adrenal Axis

**Figure 5:**
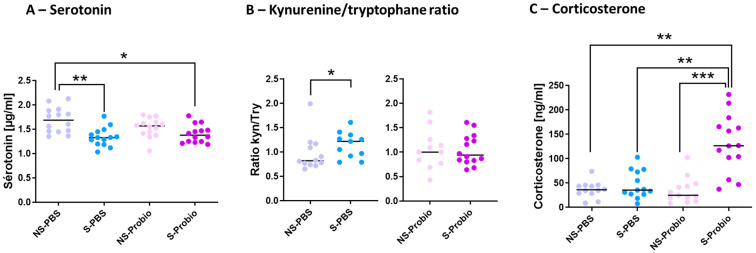
The probiotic mix following chronic restrain stress (CRS) induced changes in (**A**) serum serotonin levels, (**B**) kynurenine/tryptophane ratio and (**C**) serum corticosterone levels. Mann-Whitney and Kruskal-Wallis tests; results are expressed as median, n = 12–14. * *p* < 0.05, ** *p* < 0.01, *** *p* < 0.001. NS: non-stressed; S: stressed.

5-HT is implicated in several pathophysiological conditions, including anxiety and depression. To decipher how PM lowered the negative impact of CRS, the tryptophan (TRP) pathway was explored. The serum 5-HT concentration was significantly lower in S-PBS mice compared to NS-PBS mice (*p* < 0.01; Figure 5). This difference did not exist between S-Probio and NS-Probio mice. The serum 5-HT concentration was significantly lower in S-Probio mice than in NS-PBS mice (*p* < 0.05) (Figure 5A). Furthermore, serum kynurenine (KYN) and TRP concentrations did not differ between the four groups. Similarly, no difference was observed in the KYN/TRP ratio (Supplementary Table S2). However, when treatment effects were analysed with a Mann–Whitney test, the KYN/TRP ratio was significantly higher in S-PBS mice than in NS-PBS mice (*p* < 0.05) but not between NS-Probio and S-Probio mice (Figure 5B). Another feature of anxiety is the dysregulation of the HPA axis. No difference in the serum adrenaline concentrations was observed between groups (Supplementary Table S2). The serum corticosterone concentration measured 72 h after the last CRS exposure was significantly higher in S-Probio mice compared to all other groups (Figure 5C).

### 3.5. Differential mRNA Expression of 5-HT and Dopamine Transporters in the Hippocampus

**Figure 6:**
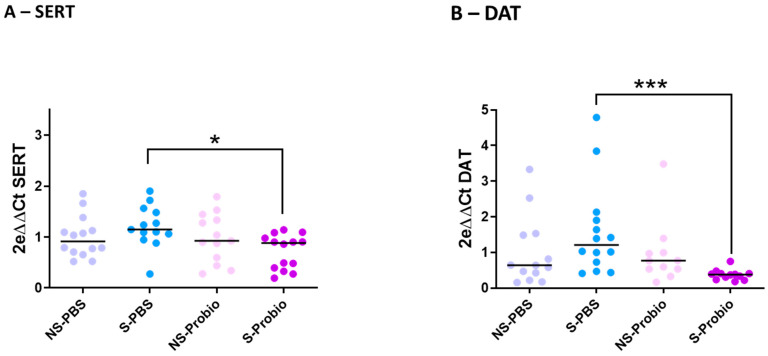
Differential mRNA expression of (**A**) serotonin (SERT) and (**B**) dopamine (DAT) transporters in the hippocampus. Kruskal-Wallis test; results are expressed as median, n = 11–14. * *p* < 0.05, *** *p* < 0.001. NS: non-stressed; S: stressed; DAT: Dopamine transporters.

To explore the mechanisms further, the expression of target genes related to stress and anxiety, monoaminergic neurotransmitters, and brain-derived neurotrophic factor (BDNF), as well as inflammation, was determined via RT-qPCR analyses in the hypothalamus, prefrontal cortex, and hippocampus. Expression of the corticotropin-releasing hormone (CRH) gene in the hypothalamus was not different between the four groups. The same was true for the expression of two other markers related to stress and anxiety, the glucocorticoid receptor (GR) and GABAa receptor (GABAaR) genes, which showed no difference between the four groups in the prefrontal cortex and hippocampus (Supplementary Table S3). Expression of the serotonergic markers TPH2 (TRP hydroxylase), 5HT1aR (5-HT receptor), and SERT (5-HT transporter) was similar between the four groups in the prefrontal cortex. The same was observed for TPH2 and 5HT1aR gene expression in the hippocampus (Supplementary Table S3). In contrast, SERT gene expression was significantly lower in S-Probio mice than in S-PBS mice (*p* < 0.05), although no impact of CRS on SERT expression was detected (Figure 6A). Expression of the TH (tyrosine hydroxylase) and DA transporter (DAT) genes, dopamine (DA) markers, showed no difference between the four groups in the prefrontal cortex. The same was true for the TH gene in the hippocampus (Supplementary Table S3). However, there was a significantly lower DAT gene expression in S-Probio mice than in S-PBS mice (*p* < 0.001), although no impact of CRS was detected on DAT expression (Figure 6B). BDNF and CREB (C-AMP Response Element-binding protein) gene expression was not significantly different in the prefrontal cortex and hippocampus between the four groups. As for the markers of inflammation, expression of the genes coding for IFNγ, TNFα, and IL-1β showed no difference in the prefrontal cortex and hippocampus between the four groups (Supplementary Table S3).

### 3.6. Expression of Target Genes Related to Inflammation and Permeability in the Ileum and Colon

**Figure 7:**
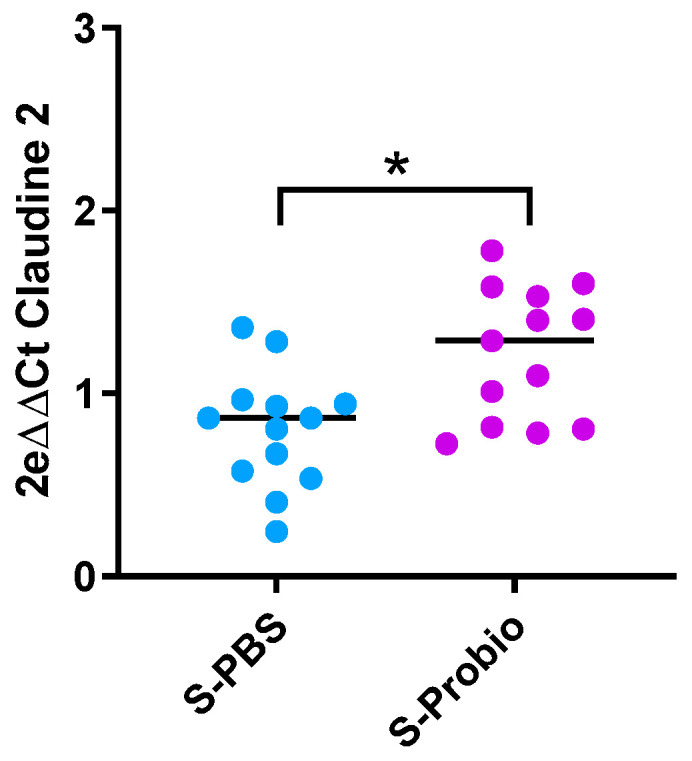
Differential mRNA expression of claudin-2 in the colon. Mann-Whitney test; results are expressed as median, n = 13. * *p* < 0.05. S: stressed.

Due to the link between anxiety and inflammation, expression of IL-1β, TNFα, and IFNγ genes was measured in the ileum and colon. There was no difference between the four groups (Supplementary Table S4). Similarly, no difference was observed in the expression of target genes related to permeability (Claudin-2, occludin, zonula occludens-1 (ZO-1), and Myosin light-chain kinase (MLCK)) in the ileum and colon. However, the *p*-value was close to significance (*p* = 0.066, Kruskal–Wallis test) for Claudin-2 in the colon (Supplementary Table S4). When the stress groups were analysed with the Mann–Whitney test, PM supplementation induced an increase in colonic Claudin-2 mRNA expression in S-Probio vs. S-PBS mice (*p* < 0.05) (Figure 7).

### 3.7. Intestinal Microbial Diversity and Composition

**Figure 8:**
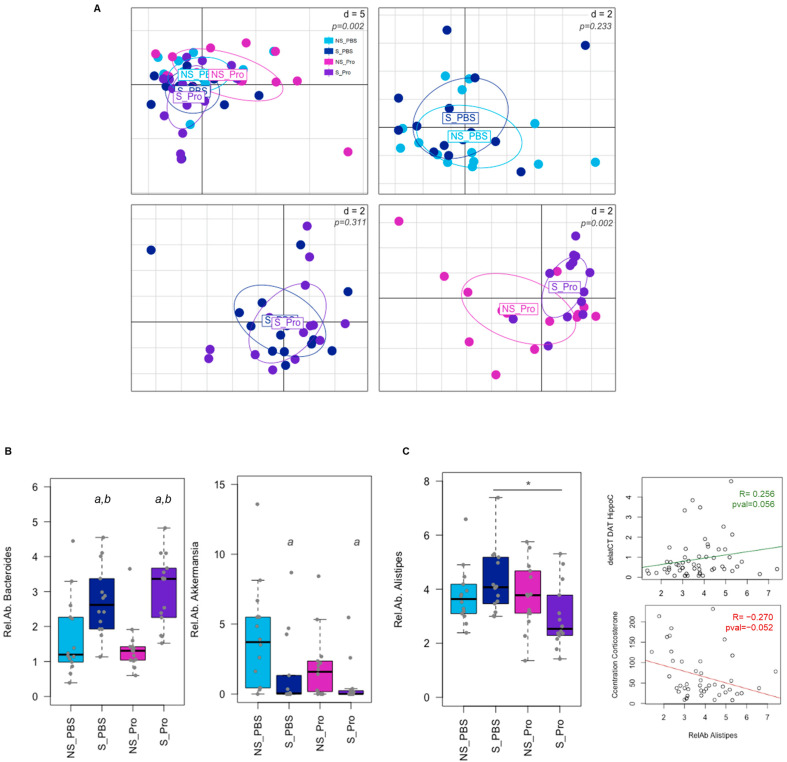
Impact of chronic restrain stress (CRS) and probiotic mix (PM) on the faecal microbiota at Week 6. (**A**) PCA of the microbial community composition at the genus level. *p*-values were simulated based on a Monte-Carlo permutation test following a bca analysis. d = distance. (**B**) Differences in bacterial taxa proportions were observed due to CRS for *Bacteroides* and *Akkermansia* genera (a: Tukey HSD *p* < 0.05 compared to NS-PBS; b: Tukey HSD *p* < 0.05 compared to NS counterparts). (**C**) Relative abundance (Rel.Ab.) of *Alistipes* genus was significantly lower in PM-treated mice under CRS (*: Tukey HSD *p* < 0.05 compared to S_PBS) and tended to correlate with biochemical modifications as indicated by the correlation lines (deltaCT DAT HippoC: expression of the DAT gene in the hippocampus; Concentration Corticosterone: serum corticosterone concentration). NS: non-stressed; S: stressed. For the lines: tended to correlate with biochemical modifications as indicated by the correlation lines.

The PM effect was assessed based on the gut microbiota of CRS mice via Illumina sequencing of the 16S rRNA. Alpha and beta diversities (at the genus level) were neither significantly impacted by CRS nor PM supplementation (Supplementary Figure S1). Analysis of faecal microbiota composition at the level of genera at 6 weeks revealed variations in the overall composition (Figure 8A). While few modifications were observed for CRS alone (NS-PBS vs. S-PBS) or PM supplementation in CRS animals (S-PBS vs. S-Probio), significant differences were detected between NS-Probio and S-Probio mice groups (Supplementary Table S5). Proportions of *Bacteroides* were significantly higher in S-Probio mice than in NS-Probio mice (Tukey HSD *p* = 0.0003 and Wilcoxon corrected *p* = 0.009). In terms of genera, CRS was associated with higher proportions of *Bacteroides* and lower proportions of *Akkermansia* (Figure 8B). PM supplementation did not appear to have any effect on this impact of stress. However, the relative abundance of the genus *Alistipes* was significantly lower in S-Probio mice than in S-PBS mice (Figure 8C). Of interest, these proportions of *Alistipes* showed a trend towards correlation with DAT gene expression in the hippocampus (*p* = 0.056, R = 0.256) and serum corticosterone concentrations (*p* = 0.052, R = −0.270) (Figure 8C).

### 3.8. Analysis of the Relative Proportions of Faecal and Caecal SCFAs

**Figure 9:**
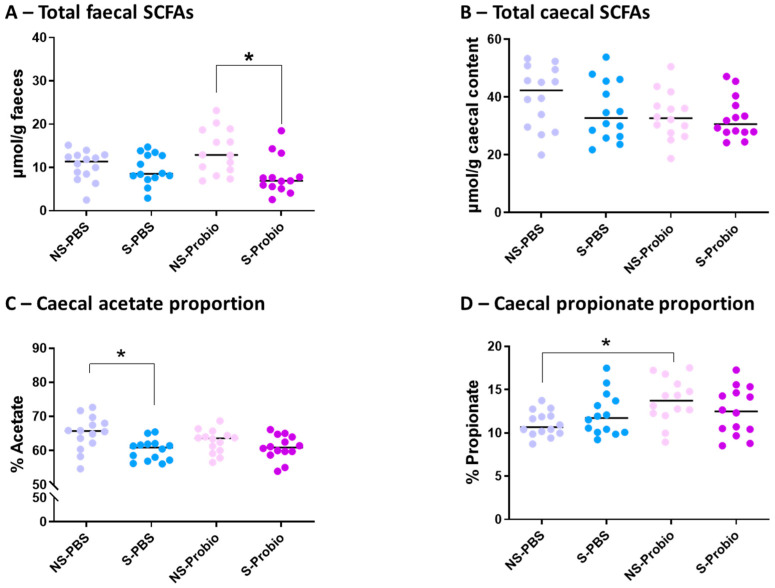
Concentration of total (**A**) faecal and (**B**) caecal short chain fatty acids (SCFAs), and proportion of caecal (**C**) acetate and (**D**) propionate. Mann-Whitney test; results are expressed as median, n = 13–14. * *p* < 0.05. NS: non-stressed; S: stressed.

No significant difference was observed in the concentration of total faecal SCFAs between S-PBS and NS-PBS mice, but a significant decrease was present in S-Probio mice compared with NS-Probio mice (*p* < 0.05) (Figure 9A). Proportions of faecal acetate, propionate, butyrate, and iso-SFCAs were not significantly different between the four groups (Supplementary Table S6). The concentration of total caecal SCFAs was not significantly different between the four groups (Figure 9B; Supplementary Table S6). However, the relative proportions of acetate in the caecal content showed that CRS was accompanied by a decrease in the proportion of acetate in S-PBS mice in comparison with their NS counterpart but not in the probiotic groups (*p* < 0.05) (Figure 9C). In addition, there was a significant increase in the proportion of propionate in NS-Probio mice compared with NS-PBS mice (*p* < 0.05) (Figure 9D).

## 4. Discussion

Millions of people live with a mental disorder; according to the WHO, in 2019, 301 million people suffered from anxiety and 280 million experienced depression [36]. Due to the adverse effects of standard treatments, such as memory impairment or addiction, the need has emerged to develop alternatives such as food supplements. In recent years, several studies have demonstrated the importance of the gut–brain axis (GBA) in these disorders. The use of probiotics to modulate GBA and improve anxiety disorders has been proposed, and the term “Psychobiotic” was defined [5]. In the present study, we assessed the effect of a mix of two probiotic strains (PM), *L. casei* LA205 and *L. paracasei* LA903, in a CRS mice model. Weekly measurements showed a reduction in weight gain as described previously after 21 or 28 days of CRS [18,37,38]. To our knowledge, coat condition had never been evaluated after CRS. However, an alteration of the coat state equivalent to our observation has been described in another chronic stress procedure, mild chronic stress [30]. These impairments in weight gain and coat condition indicate that CRS led to a degradation in the physical condition of the mice in the control group. Interestingly, even if the differences were modest, these alterations were significantly lower in S-Probio mice than in S-PBS mice, which could indicate a protective effect of PM supplementation.

In terms of behaviour, CRS was not accompanied by any alteration of performance in the OF and EPM tests under our experimental conditions. Other experiments conducted on male BALB/c or C57BL/6 mice under similar CRS conditions also reported no difference in time spent in the central area of the OF and/or the open arms of the EPM [39,40]. In contrast, several other studies showed a reduction in time spent in the OF central area and/or time spent in the EPM open arms in BALB/c mice [41,42,43]. These discrepancies could be partly explained by the use of different mouse strains, different durations of daily stress, and different experimental behavioural conditions. For instance, in [43], mice were subjected to additional stress as they received a daily i.p injection of normal saline during the CRS. Interestingly, although CRS was not associated with differences in performance in the OF and EPM tests between NS-PBS and S-PBS mice, S-Probio mice spent more time in the OF central area and made more visits in the EPM open arms than their NS counterparts. These results suggest that PM, administered before and during CRS, helped to reduce baseline anxiety and encouraged exploration of anxiogenic areas of the behavioural apparatus. This reduction in anxiety in stressed mice receiving PM could be associated with the higher serum corticosterone levels observed in these mice.

The gut microbiota is described as communicating with the brain through four main pathways (immune, neural, endocrine, and metabolic) [44]. In order to decipher how the ingested PM may exercise its protective effect, we explored all of them by focusing on targeted markers. First, we explored HPA. According to the literature, serum corticosterone levels measured after CRS depend on the time elapsed between the end of the procedure and blood sampling. Indeed, conflicting data are reported with an increase in serum corticosterone level when mice necropsy and blood sampled were performed within 24 h of the end of a CRS [41,45,46,47,48,49,50,51] and/or preceded by an additional highly stressful forced swim test [18,52]. However, serum level returned to the basal level when necropsy was delayed from the end of CRS [39,43]. We observed elevated corticosterone levels in the S-Probio group, which were significantly higher than those of NS-Probio mice but also those of stressed and unstressed mice in the PBS groups. In the absence of a higher level in NS-Probio mice, this could indicate that PM may prolong the high level of corticosterone induced by CRS rather than a direct effect of PM and could be linked to greater coping behaviour. Prolonged overproduction of glucocorticoids has been described as deleterious, particularly to cerebral function, but more recent studies suggest that glucocorticoids also confer longer-term stress-related benefits by shaping and eventually restraining stress-related physiological processes [53]. In addition, the increase remained at a physiological level compared to the one observed after stress, and recent data showed that a mild increase could protect against a greater stress response [54]. Despite the change observed in serum corticosterone, there was no change in the expression of CRH genes in the hypothalamus or GR in the prefrontal cortex and the hippocampus between the four groups. Likewise, a study showed no change in the number of hippocampus GR-immunoreactive neurons after a 6 h CRS of 21 days [39].

Neurotransmitters such as 5-HT have been widely studied in the aetiology of neuropsychiatric disorders regarding the beneficial effect of selective serotonin reuptake inhibitors on anxiety symptoms [55]. We observed that CRS led to a decrease in serum 5-HT levels in S-PBS mice in comparison with NS-PBS mice. Such a decrease in 5-HT levels was observed after a 3 h CRS for 21 days [52] but not in a 3 h CRS for 35 days [56]. In addition, Deng et al. observed, as we did, an increase in the ratio of KYN to TRP, which they attributed to a shift in serum TRP metabolism towards KYN metabolism at the detriment of 5-HT metabolism [56]. In contrast to what was seen in mice receiving PBS, those receiving PM did not display a decrease in serum 5-HT levels and an increase in the KYN /TRP ratio that followed CRS. This is the first time that a probiotic mix is reported to regulate the serum KYN/TRP ratio in a CRS model. The KYN pathway has been reported to play a role in the onset of inflammation-related mood and cognitive symptoms [57]. The blood–brain barrier is selective, and only KYN and TRP, but not 5-HT, can cross the barrier to exert effects on the homeostasis of neurotransmitters. To exercise its beneficial effect, 5-HT must, therefore, be produced directly in the brain. The enzyme responsible for TRP metabolism to 5-HT is THP2. In our study, no modification of its expression in the prefrontal cortex and hippocampus was found, suggesting an absence of 5-HT production from TRP. Although no measure of cerebral 5-HT turnover was performed, we observed a significant reduction in the main 5-HT transporter (SERT gene) in S-Probio mice compared with S-PBS mice in the hippocampus, suggesting less reuptake of 5-HT. Several studies indicate a global decrease in 5-HT levels in the brain following CRS [48,49,58]. However, this does not seem to result in the same changes in all brain regions; for example, depending on the experimental procedure, 5-HT levels did not change in the hippocampus, but decreased in the prefrontal cortex [47,56]. In addition, one study described an increase in 5-HT turnover, measured by the 5 hydroxyindoleacetic acid (5HIAA)/5-HT ratio in the hippocampus (Browne et al., 2011), but another did not [56]. Finally, a decrease in the expression of the 5-HT1A receptor has been reported in this same region [58], which was not the case in our study. Interestingly, a recent publication reported a beneficial effect of an Akkermansia strain in CRS-induced anxiety and depressive behaviours that was associated with an increase in serum 5-HT [52]. In our work, CRS was associated with lower proportions of *Akkermansia* that were not corrected by PM supplementation, suggesting that maintenance of 5-HT levels under PM supplementation may not be directly linked with *Akkermansia* properties in our model.

Dopamine (DA) is another important neurotransmitter mediating anxiety. While activation of 5HT1aR is associated with increased secretion of DA [59], no modification of TH was found in mice either in the hippocampus or in the prefrontal cortex. We only observed a significant decrease in the expression of the DAT gene in the hippocampus of S-Probio mice compared with S-PBS mice. Little is known about the repercussions of a CRS procedure on dopamine metabolism in the brain, and divergences have been reported [45,49,60]. Taken together, these data suggest that CRS favoured the reuptake of 5-HT and dopamine and that PM supplementation counteracted this, promoting the activation of the 5HT1a receptor (at an equivalent level) and the consequent coping behaviours in stressful situations.

Regarding the other markers whose gene expression was not modified in our study, we have little comparative data in the literature. For instance, for GABAergic receptors, a study showed a decrease in the gene expression of GABAα2 and GABAB1β receptors in the prefrontal cortex after a 3 h CRS for 21 days, which seems to be more linked to the forced swim test (CRS was stopped 5 days before) [18]. Although we did not observe a change in Bdnf mRNA expression in the prefrontal cortex and hippocampus after CRS, a decrease in Bdnf mRNA expression was described in the hippocampus [40,52], as well as a decrease in the BDNF protein level [56,61,62] and CREB protein level [48] in the hippocampus and prefrontal cortex.

Given the link between anxiety and neuroinflammation and the regulation of TRP pathways observed for mice supplemented with PM, we measured the expression of several key cytokines. While we did not observe any changes in their expression in the brain, two studies described an increase in the expression of two of these genes in the hippocampus, IL-1β and TNFα, but after a CRS procedure lasting 7 or 10 weeks [63,64]. Some other studies using a CRS procedure similar to ours have reported beneficial effects of probiotics on proinflammatory markers in brains [41,46,61]. It has been described that the source of neuroinflammation could be intestinal permeability [65]. Gene expression was studied in the ileum and colon, but only an increase in the expression of Claudin-2 mRNA was observed in S-Probio mice compared to S-PBS mice. Proteins from the claudin family are necessary components of tight junctions, and Claudin 2 is specifically involved in the formation of pores that allow paracellular diffusion of small molecules [66]. However, gene modification of a single marker makes interpretation difficult. While we saw no difference in PBS mice exposed to CRS, a study observed a decrease in the expression of ZO-1, Claudin, and occludin genes following a 3 h CRS for 14 days, which was considered to reflect an alteration of the gut barrier [50]. Similar to us, two studies investigating a broad set of markers of inflammation in the colon, including those we measured, found no differences in stressed mice in comparison to controls [18,49]. Only one study, using a lengthy CRS of 10 weeks, described an increase in TNFα and IL-1β in the colon [64]. In addition, two studies reported lower systemic and/or intestinal inflammation after supplementation with *Bifidobacterium pseudocatenulatum* CECT 7765 and *L. paracasei* PS23 strains, but they used an early maternal separation stress-induced model [15,67]. Altogether, these data indicate that in our experimental model, neither inflammation nor leaky gut was associated with the CRS-induced phenotype.

Published data indicate an altered microbiota in psychiatric disorders, including anxiety. However, a recent systematic review of patients suffering from major depression did not report consistent results on microbiota diversity and composition after probiotic intervention, indicating that the benefits of probiotics could be due to other mechanisms, such as secreted metabolites [68]. While neither alpha nor beta diversity was significantly altered, CRS was associated with a higher relative abundance of *Bacteroides* and a lower proportion of *Akkermansia*. Yet, PM was not able to counteract these modifications. To our knowledge, we are the first to address this feature in the BALB/c genetic model. Indeed, studies mainly reported an impact of CRS on the gut microbiota in C57BL6 mice with few differences in bacterial communities due to different experimental designs and types of collected samples (caecal vs. faeces) [56,64,69]. By contrast, we noted a lower relative abundance of *Alistipes* in the PM-supplemented mice compared to the PBS-supplemented mice exposed to CRS. It is interesting to note that *Alistipes* has been associated with stress in BALB/c mice [70], and a clinical study revealed an increase in this genus in patients suffering from depression compared to healthy patients [71].

In mice that received PM, the only change we observed in the faeces was a reduction in the total amount of SFCAs in S-Probio mice compared with NS-Probio mice, a change that did not exist in caecal contents. The total level of SFCAs measured in the faeces and caecal contents were not modified by the CRS in PBS mice. One study described a reduction in the total level of SFCAs in the faeces, but this was after a 10-week CRS [64]. The caecal acetate proportion was decreased in S-PBS mice in comparison to NS-PBS mice; this is in line with the decrease in acetate levels described after a 4 h CRS for 28 days (Wu et al., 2020). However, it should be noted that these authors also observed a reduction in caecal propionate and butyrate levels, but no difference was described for all of the SCFAs in the faeces and caecum content after a 3 h CRS for 14 days [50]. Among the main acetate producers, the abundance of the *Akkermansia* genus was reduced in the present study after CRS, which could, in part, explain the lower acetate level. The decrease in the proportion of caecal acetate observed in PBS-supplemented mice did not exist in PM-supplemented mice. This could be due to a regulating effect of PM. However, PM had an effect on the caecal proportion of propionate in unstressed mice, indicating a specific effect of the mix in the absence of stress.

We noted a few limitations to our study. Firstly, we only used male mice, and it is known that stress has a sex-dependent effect. However, Meng et al. [40] described a higher effect of CRS in females in their model, so we would expect our observations to be observed in females as well. In addition, to assess the mechanism, we mainly focused on mRNA expression, which will require validation at the protein level. However, we found consistencies between the serum level and brain regulation for 5-HT. In addition, we focused on anxiety symptoms, but due to the comorbidity existing between anxiety and depression, it will be interesting to address the depressive symptoms with other behavioural tests assessing helplessness, such as forced swim or tail suspension tests, or anhaedonia, such as the sucrose preference test. Finally, although our work contributes to providing information on the gut–brain axis, this study was performed on animals, and it remains necessary to investigate the effect of probiotics on humans.

## 5. Conclusions

We demonstrated that supplementation with a mix of two probiotic strains, *L. casei* LA205 and *L. paracasei* LA903, helped animals to cope better with CRS (i.e., loss of body weight and improvement in coat state) and a new stressful situation, as shown by behavioural tests, with mice spending more time in the centre in OF and open arms in EPM. PM reduced 5-HT and DA reuptake in the hippocampus, increased the gene expression of Claudin-2, and counteracted the drop in serum 5-HT and the higher Kynurenine/TRP ratio observed in CRS-induced mice. The absence of major modifications of the faecal microbiota, with the exception of a lower abundance of *Akkermansia,* potentially responsible for the decrease in acetate levels, suggested that PM mainly act directly, even if the precise mechanism is still under investigation. PM-supplemented mice exhibited a lower *Alistipes* abundance associated with depressive symptoms. Altogether, these results contribute to the understanding of the effects of probiotics in CRS models via the gut–brain axis.

## Figures and Tables

**Table 1:** Primers used for qPCR with the cDNA solution obtained from brain tissue (TaqMan™ Gene Expression Assay (Applied Biosystems, Fisher Scientific)).

Marker	Reference	Dye	Marker	Reference	Dye
IL-1β	Mm00434228_m1	FAM	BDNF	Mm04230607_m1	VIC
TNFα	Mm00443258_m1	FAM	CREB	Mm01342452_m1	VIC
TH	Mm00447557_m1	FAM	GR	Mm00433832_m1	VIC
GAPDH	Mm99999915_g1	FAM	TPH2	Mm00557722_m1	VIC
IFNγ	Mm01168134_m1	FAM	5HT1aR	Mm00434106_m1	VIC
DAT	Mm00438368_m1	FAM	GABAaR	Mm00433489_m1	VIC
SERT	Mm00439391_m1	FAM	Actine b	Mm02619580_g1	VIC

IL-1β, Interleukin 1 Beta; TNFα, Tumor necrosis factor alpha; TH, Tyrosine hydroxylase; GAPDH, Glyceraldehyde-3-phosphate Dehydrogenase; IFNγ, Interferon gamma; DAT, Dopamine transporter; SERT, serotonin transporter; BDNF, Brain-derived neurotrophic factor; CREB, Cyclic AMP Responsive Element Binding; GR, Glucocorticoid receptor; TPH2, Tryptophan hydroxylase 2; 5HT1aR, serotonin 1A receptor; GABAaR, Gamma-aminobutyric acid a receptor; Actine b, Actine beta.

**Table 2:** Primers used for qPCR with the cDNA solution obtained from intestine tissue (SYBR™ Green PCR Master Mix (Applied Biosystems, Fisher Scientific)).

Probes	Forward	Reverse
β-actine	5′ CTAAGGCCAACCGTG 3′	5′ ACCAGAGGCATACAG 3′
IFNγ	5′ ATCTGGAGGAACTGGCAAAA 3′	5′ TTCAAGACTTCAAAGAGTCTGAGGTA 3′
Occludine	5′ GCGATCATACCCAGAGTCTTTC 3′	5′ TGCCTGAAGTCATCCACACT 3′
TNFα	5′ CTGTAGCCCACGTCGTAGC 3′	5′ TTGAGATCCATGCCGTTG 3′
MLCK	5′ TGGTAGGGTCCACATTCCAG 3′	5′ CTGCTTGCTCCTTGTTCTCC 3′
IL-1β	5′ AGTTGACGGACCCAAAAG 3′	5′ AGCTGGATGCTCTCATCAGG 3′
ZO-1	5′ GATCATTCCACGCAGTCTCC 34	5′ GGCCCCAGGTTTAGACATTC 3′
Claudine 2	5′ GTAGCCGGAGTCATCCTTTG 3′	5′ GGCCTGGTAGCCATCATAGT 3′

## Data Availability

Microbiome data related to 16S sequence analyses of all samples will be made available upon publication acceptance under SRA BioProject, ID PRJNA998594. All other relevant data may be accessible upon reasonable request to the corresponding author.

## Supplementary Materials

The following supporting information can be downloaded at: https://www.mdpi.com/article/10.3390/nu15214635/s1, Figure S1: Overtime impact of PM supplementation in mice under CRS; Table S1: Kruskal-Wallis test analysis of the results obtained in the OF and EPM tests; Table S2: Comparison of blood markers in serum using the Kruskal-Wallis test; Table S3: Comparison of brain markers using the Kruskal-Wallis test; Table S4: Comparison of inflammation and gut permeability using the Kruskal-Wallis test; Table S5: Relative abundance significantly regulated between treatments at week 6; Table S6: Comparison of total SCFAS and percentages of acetate, propionate, butyrate and iso-SCFAs using the Kruskal-Wallis test.

## References

[1] (2022). Mental Health and COVID-19: Early Evidence of the Pandemic’s Impact.

[2] Barchielli B., Cricenti C., Gallè F., Sabella E.A., Liguori F., Da Molin G., Liguori G., Orsi G.B., Giannini A.M., Ferracuti S. (2022). Climate Changes, Natural Resources Depletion, COVID-19 Pandemic, and Russian-Ukrainian War: What Is the Impact on Habits Change and Mental Health?. Int. J. Environ. Res. Public Health.

[3] Dudek K.A., Dion-Albert L., Lebel M., LeClair K., Labrecque S., Tuck E., Ferrer Perez C., Golden S.A., Tamminga C., Turecki G. (2020). Molecular adaptations of the blood-brain barrier promote stress resilience vs. depression. Proc. Natl Acad. Sci. USA.

[4] Marcolongo-Pereira C., Castro F.C.A.Q., Barcelos R.M., Chiepe K.C.M.B., Rossoni Junior J.V., Ambrosio R.P., Chiarelli-Neto O., Pesarico A.P. (2022). Neurobiological mechanisms of mood disorders: Stress vulnerability and resilience. Front. Behav. Neurosci..

[5] Dinan T.G., Stanton C., Cryan J.F. (2013). Psychobiotics: A novel class of psychotropic. Biol. Psychiatry.

[6] Collins S.M., Bercik P. (2009). The relationship between intestinal microbiota and the central nervous system in normal gastrointestinal function and disease. Gastrenterology.

[7] Crumeyrolle-Arias M., Jaglin M., Bruneau A., Vancassel S., Cardona A., Daugé V., Naudon L., Rabot S. (2014). Absence of the gut microbiota enhances anxiety-like behavior and neuroendocrine response to acute stress in rats. Psychoneuroendocrinology.

[8] Sudo N., Chida Y., Aiba Y., Sonoda J., Oyama N., Yu X.N., Kubo C., Koga Y. (2004). Postnatal microbial colonization programs the hypothalamic-pituitary-adrenal system for stress response in mice. J. Physiol..

[9] Cryan J.F., Dinan T.G. (2012). Mind-altering microorganisms: The impact of the gut microbiota on brain and behaviour. Nat. Rev. Neurosci..

[10] Chevalier G., Siopi E., Guenin-Macé L., Pascal M., Laval T., Rifflet A., Boneca I.G., Demangel C., Colsch B., Pruvost A. (2020). Effect of gut microbiota on depressive-like behaviors in mice is mediated by the endocannabinoid system. Nat. Commun..

[11] Li N., Wang Q., Wang Y., Sun A., Lin Y., Jin Y., Li X. (2019). Fecal microbiota transplantation from chronic unpredictable mild stress mice donors affects anxiety-like and depression-like behavior in recipient mice via the gut microbiota-inflammation-brain axis. Stress.

[12] Mir H.D., Milman A., Monnoye M., Douard V., Philippe C., Aubert A., Castanon N., Vancassel S., Guérineau N.C., Naudon L. (2020). The gut microbiota metabolite indole increases emotional responses and adrenal medulla activity in chronically stressed male mice. Psychoneuroendocrinology.

[13] Bravo J.A., Forsythe P., Chew M.V., Escaravage E., Savignac H.M., Dinan T.G., Bienenstock J., Cryan J.F. (2011). Ingestion of Lactobacillus strain regulates emotional behavior and central GABA receptor expression in a mouse via the vagus nerve. Proc. Natl Acad. Sci. USA.

[14] Dhaliwal J., Singh D.P., Singh S., Pinnaka A.K., Boparai R.K., Bishnoi M., Kondepudi K.K., Chopra K. (2018). Lactobacillus plantarum MTCC 9510 supplementation protects from chronic unpredictable and sleep deprivation-induced behaviour, biochemical and selected gut microbial aberrations in mice. J. Appl. Microbiol..

[15] Liao J.F., Hsu C.C., Chou G.T., Hsu J.S., Liong M.T., Tsai Y.C. (2019). Lactobacillus paracasei PS23 reduced early-life stress abnormalities in maternal separation mouse model. Benef. Microbes.

[16] Liu Y.W., Liu W.H., Wu C.C., Juan Y.C., Wu Y.C., Tsai H.P., Wang S., Tsai Y.C. (2016). Psychotropic effects of Lactobacillus plantarum PS128 in early life-stressed and naïve adult mice. Brain Res..

[17] Maehata H., Kobayashi Y., Mitsuyama E., Kawase T., Kuhara T., Xiao J.Z., Tsukahara T., Toyoda A. (2019). Heat-killed Lactobacillus helveticus strain MCC1848 confers resilience to anxiety or depression-like symptoms caused by subchronic social defeat stress in mice. Biosci. Biotechnol. Biochem..

[18] Stenman L.K., Patterson E., Meunier J., Roman F.J., Lehtinen M.J. (2020). Strain specific stress-modulating effects of candidate probiotics: A systematic screening in a mouse model of chronic restraint stress. Behav. Brain Res..

[19] Xu M., Tian P., Zhu H., Zou R., Zhao J., Zhang H., Wang G., Chen W. (2022). Lactobacillus paracasei CCFM1229 and Lactobacillus rhamnosus CCFM1228 Alleviated Depression- and Anxiety-Related Symptoms of Chronic Stress-Induced Depression in Mice by Regulating Xanthine Oxidase Activity in the Brain. Nutrients.

[20] Chong H.X., Yusoff N.A.A., Hor Y.Y., Lew L.C., Jaafar M.H., Choi S.B., Yusoff M.S.B., Wahid N., Abdullah M.F.I.L., Zakaria N. (2019). Lactobacillus plantarum DR7 alleviates stress and anxiety in adults: A randomised, double-blind, placebo-controlled study. Benef. Microbes.

[21] Kato-Kataoka A., Nishida K., Takada M., Suda K., Kawai M., Shimizu K., Kushiro A., Hoshi R., Watanabe O., Igarashi T. (2016). Fermented milk containing Lactobacillus casei strain Shirota prevents the onset of physical symptoms in medical students under academic examination stress. Benef. Microbes.

[22] Lew L.C., Hor Y.Y., Yusoff N.A.A., Choi S.B., Yusoff M.S.B., Roslan N.S., Ahmad A., Mohammad J.A.M., Abdullah M.F.I.L., Zakaria N. (2019). Probiotic Lactobacillus plantarum P8 alleviated stress and anxiety while enhancing memory and cognition in stressed adults: A randomised, double-blind; placebo-controlled study. Clin. Nutr..

[23] Nishida K., Sawada D., Kuwano Y., Tanaka H., Rokutan K. (2019). Health Benefits of Lactobacillus gasseri CP2305 Tablets in Young Adults Exposed to Chronic Stress: A Randomized, Double-Blind, Placebo-Controlled Study. Nutrients.

[24] Patterson E., Griffin S.M., Ibarra A., Ellsiepen E., Hellhammer J. (2020). Lacticaseibacillus paracasei Lpc-37^®^ improves psychological and physiological markers of stress and anxiety in healthy adults: A randomized, double-blind; placebo-controlled and parallel clinical trial (the Sisu study). Neurobiol. Stress.

[25] Wauters L., Van Oudenhove L., Accarie A., Geboers K., Geysen H., Toth J., Luypaerts A., Verbeke K., Smokvina T., Raes J. (2022). Lactobacillus rhamnosus CNCM I-3690 decreases subjective academic stress in healthy adults: A randomized placebo-controlled trial. Gut Microbes.

[26] Le Morvan de Sequeira C., Hengstberger C., Enck P., Mack I. (2022). Effect of Probiotics on Psychiatric Symptoms and Central Nervous System Functions in Human Health and Disease: A Systematic Review and Meta-Analysis. Nutrients.

[27] Ng Q.X., Peters C., Ho C.Y.X., Lim D.Y., Yeo W.S. (2018). A meta-analysis of the use of probiotics to alleviate depressive symptoms. J. Affect. Disord..

[28] Belzung C., Griebel G. (2001). Measuring normal and pathological anxiety-like behaviour in mice: A review. Behav. Brain Res..

[29] Becker M., Pinhasov A., Ornoy A. (2021). Animal Models of Depression: What Can They Teach Us about the Human Disease?. Diagnostics.

[30] Yalcin I., Aksu F., Belzung C. (2005). Effects of desipramine and tramadol in a chronic mild stress model in mice are altered by yohimbine but not by pindolol. Eur. J. Pharmacol..

[31] Prut L., Belzung C. (2003). The open field as a paradigm to measure the effects of drugs on anxiety-like behaviors: A review. Eur. J. Pharmacol..

[32] Jaglin M., Rhimi M., Philippe C., Pons N., Bruneau A., Goustard B., Daugé V., Maguin E., Naudon L., Rabot S. (2018). Indole, a signaling molecule produced by the gut microbiota, negatively impacts emotional behaviors in rats. Front. Neurosci..

[33] Callahan B.J., McMurdie P.J., Rosen M.J., Han A.W., Johnson A.J.A., Holmes S.P. (2016). DADA2: High-resolution sample inference from Illumina amplicon data. Nat. Methods.

[34] Escudié F., Auer L., Bernard M., Mariadassou M., Cauquil L., Vidal K., Maman S., Hernandez-Raquet G., Combes S., Pascal G. (2018). FROGS: Find, Rapidly, OTUs with Galaxy Solution. Bioinformatics.

[35] Rabot S., Szylit O., Nugon-Baudon L., Meslin J.C., Vaissade P., Popot F., Viso M. (2000). Variations in digestive physiology of rats after short duration flights aboard the US space shuttle. Dig. Dis. Sci..

[36] WHO. https://www.who.int/news-room/fact-sheets/detail/mental-disorders.

[37] Tian T., Mao Q., Xie J., Wang Y., Shao W.H., Zhong Q., Chen J.J. (2022). Multi-omics data reveals the disturbance of glycerophospholipid metabolism caused by disordered gut microbiota in depressed mice. J. Adv. Res..

[38] Wu M., Tian T., Mao Q., Zou T., Zhou C.J., Xie J., Chen J.J. (2020). Associations between disordered gut microbiota and changes of neurotransmitters and short-chain fatty acids in depressed mice. Transl. Psychiatry.

[39] Liu X., Wu R., Tai F., Ma L., Wei B., Yang X., Zhang X., Jia R. (2013). Effects of group housing on stress induced emotional and neuroendocrine alterations. Brain Res..

[40] Meng F., Liu J., Dai J., Lian H., Jiang S., Li Q., Wu M., Wang W., Wang D., Zhao D. (2021). PPM1F in Dentate Gyrus Modulates Anxiety-Related Behaviors by Regulating BDNF Expression via AKT/JNK/p-H3S10 Pathway. Mol. Neurobiol..

[41] Banagozar Mohammadi A., Torbati M., Farajdokht F., Sadigh-Eteghad S., Fazljou S.M.B., Vatandoust S.M., Golzari S.E.J., Mahmoudi J. (2019). Sericin alleviates restraint stress induced depressive- and anxiety-like behaviors via modulation of oxidative stress, neuroinflammation and apoptosis in the prefrontal cortex and hippocampus. Brain Res..

[42] Ou F., Su K., Sun J., Zhang Z., Peng Y., Liao G. (2019). Temporomandibular joint disorders contribute to anxiety in BalB/C mice. Biochem. Biophys. Res. Commun..

[43] Salehi A., Rabiei Z., Setorki M. (2018). Effect of gallic acid on chronic restraint stress-induced anxiety and memory loss in male BALB/c mice. Iran J. Basic Med. Sci..

[44] Kennedy P.J., Cryan J.F., Dinan T.G., Clarke G. (2017). Kynurenine pathway metabolism and the microbiota-gut-brain axis. Neuropharmacology.

[45] Browne C.A., Clarke G., Dinan T.G., Cryan J.F. (2011). Differential stress-induced alterations in tryptophan hydroxylase activity and serotonin turnover in two inbred mouse strains. Neuropharmacology.

[46] Kwatra M., Ahmed S., Gangipangi V.K., Panda S.R., Gupta N., Shantanu P.A., Gawali B., Naidu V.G.M. (2021). Lipopolysaccharide exacerbates chronic restraint stress-induced neurobehavioral deficits: Mechanisms by redox imbalance, ASK1-related apoptosis, autophagic dysregulation. J. Psychiatr. Res..

[47] Oh D.R., Kim Y., Choi E.J., Jung M.A., Oh K.N., Hong J.A., Bae D., Kim K., Kang H., Kim J. (2018). Antidepressant-Like Effects of Vaccinium bracteatum in Chronic Restraint Stress Mice: Functional Actions and Mechanism Explorations. Am. J. Chin. Med..

[48] Park S.H., Jang S., Lee S.W., Park S.D., Sung Y.Y., Kim H.K. (2018). Akebia quinata Decaisne aqueous extract acts as a novel anti-fatigue agent in mice exposed to chronic restraint stress. J. Ethnopharmacol..

[49] Puppala E.R., Aochenlar S.L., Shantanu P.A., Ahmed S., Jannu A.K., Jala A., Yalamarthi S.S., Borkar R.M., Tripathi D.M., Naidu V.G.M. (2022). Perillyl alcohol attenuates chronic restraint stress aggravated dextran sulfate sodium-induced ulcerative colitis by modulating TLR4/NF-κB and JAK2/STAT3 signaling pathways. Phytomedicine.

[50] Tian P., Zhu H., Qian X., Chen Y., Wang Z., Zhao J., Zhang H., Wang G., Chen W. (2021). Consumption of Butylated Starch Alleviates the Chronic Restraint Stress-Induced Neurobehavioral and Gut Barrier Deficits Through Reshaping the Gut Microbiota. Front. Immunol..

[51] Wanasuntronwong A., Tantisira M.H., Tantisira B., Watanabe H. (2012). Anxiolytic effects of standardized extract of Centella asiatica (ECa 233) after chronic immobilization stress in mice. J. Ethnopharmacol..

[52] Ding Y., Bu F., Chen T., Shi G., Yuan X., Feng Z., Duan Z., Wang R., Zhang S., Wang Q. (2021). A next-generation probiotic: Akkermansia muciniphila ameliorates chronic stress-induced depressive-like behavior in mice by regulating gut microbiota and metabolites. Appl. Microbiol. Biotechnol..

[53] Raison C.L., Miller A.H. (2003). When not enough is too much: The role of insufficient glucocorticoid signaling in the pathophysiology of stress-related disorders. Am. J. Psychiatry.

[54] Dinel A.L., Guinobert I., Lucas C., Blondeau C., Bardot V., Ripoche I., Berthomier L., Pallet V., Layé S., Joffre C. (2019). Reduction of acute mild stress corticosterone response and changes in stress-responsive gene expression in male Balb/c mice after repeated administration of a Rhodiola rosea L. root extract. Food Sci. Nutr..

[55] Huang F., Wu X. (2021). Brain Neurotransmitter Modulation by Gut Microbiota in Anxiety and Depression. Front. Cell Dev. Biol..

[56] Deng Y., Zhou M., Wang J., Yao J., Yu J., Liu W., Wu L., Wang J., Gao R. (2021). Involvement of the microbiota-gut-brain axis in chronic restraint stress: Disturbances of the kynurenine metabolic pathway in both the gut and brain. Gut Microbes.

[57] Capuron L., Castanon N. (2017). Role of Inflammation in the Development of Neuropsychiatric Symptom Domains: Evidence and Mechanisms. Curr. Top. Behav. Neurosci..

[58] Liao S., Lv J., Zhou J., Kalavagunta P.K., Shang J. (2017). Effects of two chronic stresses on mental state and hair follicle melanogenesis in mice. Exp. Dermatol..

[59] Ichikawa J., Ishii H., Bonaccorso S., Fowler W.L., O’Laughlin I.A., Meltzer H.Y. (2001). 5-HT(2A) and D(2) receptor blockade increases cortical DA release via 5-HT(1A) receptor activation: A possible mechanism of atypical antipsychotic-induced cortical dopamine release. J. Neurochem..

[60] Sahoo S., Kharkar P.S., Sahu N.U. (2021). Anxiolytic activity of Psidium guajava in mice subjected to chronic restraint stress and effect on neurotransmitters in brain. Phytother. Res..

[61] Guo Y., Xie J.P., Deng K., Li X., Yuan Y., Xuan Q., Xie J., He X.M., Wang Q., Li J.J. (2019). Prophylactic Effects of Bifidobacterium adolescentis on Anxiety and Depression-Like Phenotypes After Chronic Stress: A Role of the Gut Microbiota-Inflammation Axis. Front. Behav. Neurosci.

[62] Sun X., Zhang H.F., Ma C.L., Wei H., Li B.M., Luo J. (2021). Alleviation of Anxiety/Depressive-Like Behaviors and Improvement of Cognitive Functions by Lactobacillus plantarum WLPL04 in Chronically Stressed Mice. Can. J. Infect. Dis. Med. Microbiol..

[63] Liu H., Liu L.L., Chen J., Chen Y.W., Chai Y., Liu Q.S., Cheng Y. (2022). Muscone with Attenuation of Neuroinflammation and Oxidative Stress Exerts Antidepressant-Like Effect in Mouse Model of Chronic Restraint Stress. Oxid. Med. Cell. Longev..

[64] Xiao Q., Shu R., Wu C., Tong Y., Xiong Z., Zhou J., Yu C., Xie X., Fu Z. (2020). Crocin-I alleviates the depression-like behaviors probably via modulating “microbiota-gut-brain” axis in mice exposed to chronic restraint stress. J. Affect. Disord..

[65] Kelly J.R., Kennedy P.J., Cryan J.F., Dinan T.G., Clarke G., Hyland N.P. (2015). Breaking down the barriers: The gut microbiome, intestinal permeability and stress-related psychiatric disorders. Front Cell Neurosci..

[66] Zhang X. (2018). The Role of Dietary Sugars in Intestinal Homeostasis and Microbiota Composition. Ph.D. Thesis.

[67] Moya-Pérez A., Perez-Villalba A., Benítez-Páez A., Campillo I., Sanz Y. (2017). Bifidobacterium CECT 7765 modulates early stress-induced immune; neuroendocrine and behavioral alterations in mice. Brain Behav. Immun..

[68] Ng Q.X., Lim Y.L., Yaow C.Y.L., Ng W.K., Thumboo J., Liew T.M. (2023). Effect of Probiotic Supplementation on Gut Microbiota in Patients with Major Depressive Disorders: A Systematic Review. Nutrients.

[69] Zhuang Y., Zeng R., Liu X., Yang L., Chan Z. (2022). Neoagaro-Oligosaccharides Ameliorate Chronic Restraint Stress-Induced Depression by Increasing 5-HT and BDNF in the Brain and Remodeling the Gut Microbiota of Mice. Mar. Drugs.

[70] Bangsgaard Bendtsen K.M., Krych L., Sørensen D.B., Pang W., Nielsen D.S., Josefsen K., Hansen L.H., Sørensen S.J., Hansen A.K. (2012). Gut microbiota composition is correlated to grid floor induced stress and behavior in the BALB/c mouse. PLoS ONE.

[71] Jiang H., Ling Z., Zhang Y., Mao H., Ma Z., Yin Y., Wang W., Tang W., Tan Z., Shi J. (2015). Altered fecal microbiota composition in patients with major depressive disorder. Brain Behav. Immun..

