# Supplementary material for: Effects of a Lacticaseibacillus Mix on Behavioural, Biochemical, and Gut Microbial Outcomes of Male Mice following Chronic Restraint Stress
Source: Nutrients. 2023 Oct 31;15(21):4635. doi: 10.3390/nu15214635 (PMC10648220; doi:10.3390/nu15214635)
Supplement: Supplementary file 1 [file nutrients-15-04635-s001.zip › Supplementary tables Letenneur et al..pdf]

Tests	Parameters		NS-PBS	S-PBS	NS-Probio	S-Probio	Kruskal-Wallis test P value
OF	Number of visits	Periphery	39.00 (14.00)	34.50 (27.25)	37.50 (12.50)	42.00 (16.00)	0.339
		Centre	38.50 (14.00)	33.50 (28.25)	37.00 (12.50)	41.00 (16.25)	0.369
		Corners	58.00 (16.50)	59.50 (12.75)	57.00 (12.75)	58.00 (11.75)	0.932
	Time (s)	Periphery	306.90 (34.30)	310.10 (46.20)	310.10 (15.90)	296.70 (40.00)	0.374
		Centre	53.15 (34.30)	49.95 (46.20)	49.30 (28.08)	63.30 (40.00)	0.324
		Corners	112.1 (39.32)	110.6 (60.02)	112.2 (40.50)	98.90 (36.50)	0.305
	Total distance (m)		26.70 (5.46)	26.41 (6.90)	26.43 (9.94)	27.02 (7.26)	0.992
	Rearing		39.00 (21.50)	20.00 (26.50)	29.50 (36.25)	14.50 (36.50)	0.108
	Grooming		1.00 (1.75)	1.00 (0.25)	1.00 (1.00)	1.00 (2.00)	0.604
	Faeces		6.50 (2.75)	7.00 (3.00)	6.00 (5.00)	6.50 (4.50)	0.766
EPM	Number of visits	% open arms	57.50 (18.75)	60.50 (33.75)	56.00 (21.25)	71.00 (15.00)	0.164
		Centre	36.50 (9.25)	30.00 (7.00)	35.50 (9.75)	32.00 (18.00)	0.064
		End of the arms	13.00 (10.75)	11.50 (4.75)	7.50 (10.00)	11.00 (8.50)	0.366
	Time (s)	% open arms	65.05 (24.55)	68.85 (22.25)	54.85 (39.55)	72.00 (22.60)	0.422
		Centre	90.75 (42.10)	60.20 (24.75)	84.10 (49.30)	56.90 (60.65)	0.267
		End of the arms	35.55 (54.00)	55.35 (38.87)	21.60 (22.21)	36.20 (58.15)	0.058
	Head bents	Centre	5.00 (5.50)	2.00 (5.00)	4.50 (7.00)	4.00 (5.50)	0.285
		Open arms	6.50 (5.50)	5.00 (13.00)	7.00 (10.50)	8.00 (11.50)	0.761
	Faeces		4.00 (3.75)	5.50 (4.00)	4.00 (2.50)	5.00 (2.50)	0.699

Table S1 : Kruskal-Wallis test analysis of the results obtained in the OF and EPM tests. Results are expressed as median (interquartile range), n = 12-14.

	NS-PBS	S-PBS	NS-Probio	S-Probio	Kruskal-Wallis test P value
Serotonin (µg/ml)	1.7 (0.4)	1.3 (0.3)	1.6 (0.3)	1.4 (0.3)	0.0009
Kynurenin (ng/ml)	177.6 (151.3)	228.5 (90.8)	197.1 (100.4)	227.7 (121.5)	0.70
Tryptophan (µg/ml)	22.6 (10.6)	20.3 (8.5)	20.3 (11.3)	21.3 (11.7)	0.62
Kynurenin/Tryptophan	0.8 (0.4)	1.2 (0.4)	1.0 (0.5)	0.9 (0.5)	0.29
Adrenaline (µg/ml)	3.3 (2.3)	2.0 (1.4)	2.6 (1.3)	2.7 (2.0)	0.11
Corticosterone (ng/ml)	36.1 (13.5)	35.5 (48.4)	24.9 (29.9)	126.7 (79.4)	< 0.0001

Table S2 : Comparison of blood markers in serum using the Kruskal-Wallis test. Results are expressed as median (interquartile range), n = 11-14.

Brain regions	Marker	NS-PBS	S-PBS	NS-Probio	S-Probio	Kruskal-Wallis test P value
Hypothalamus	CRH	0.9 (0. 6)	1.2 (0.4)	1.0 (0.4)	1.2 (0.5)	0.66
Prefrontal cortex	GR	1.0 (0.4)	1.0 (0.2)	1.0 (0.3)	1.0 (0.1)	0.54
	GABAaR	1.0 (0.3)	1.1 (0.3)	1.1 (0.3)	1.1 (0.1)	0.66
	TPH2	1.1 (0.4)	1.1 (0.3)	1.1 (0.5)	0.9 (0.2)	0.50
	5HT1AR	1.0 (0.4)	0.9 (0.3)	1.0 (0.4)	0.9 (0.3)	0.78
	SERT	1.1 (0.3)	1.3 (0.5)	1.3 (0.8)	1.0 (0.4)	0.24
	TH	1.1 (1.4)	0.7 (2.1)	0.5 (0.8)	0.3 (1.3)	0.51
	DAT	1.0 (1.3)	0.7 (2.1)	0.4 (0.7)	0.3 (1.3)	0.41
	BDNF	1.1 (0.6)	1.1 (0.7)	0.9 (0.4)	1.0 (0.5)	0.55
	CREB	1.1 (0.3)	1.0 (0.1)	0.9 (0.3)	1.0 (0.3)	0.14
	IFNγ	0.9 (0.7)	0.8 (1.0)	1.0 (0.7)	0.5 (0.7)	0.19
	IL-1β	0.9 (0.4)	1.0 (0.3)	1.2 (0.8)	1.0 (0.4)	0.79
	TNFα	1.1 (0.4)	1.0 (0.6)	0.8 (0.2)	0.9 (0.7)	0.35
Hippocampus	GR	0.9 (0.4)	0.8 (0.3)	0.9 (0.2)	0.9 (0.4)	0.73
	GABAaR	1.0 (0.2)	1.0 (0.3)	1.0 (0.4)	1.0 (0.5)	0.90
	TPH2	1.0 (0.2)	1.1 (0.2)	1.0 (0.3)	1.0 (0.3)	0.10

	5HT1AR	1.0 (0.6)	1.2 (0.6)	1.2 (0.6)	1.1 (0.5)	0.50
	SERT	1.0 (0.5)	1.1 (0.5)	1.0 (0.9)	0.9 (0.6)	0.03
	TH	0.9 (0.7)	0.9 (0.5)	0.9 (1.0)	0.9 (0.6)	0.97
	DAT	0.6 (1.2)	1.2 (1.3)	0.8 (0.5)	0.4 (0.1)	0.0009
	BDNF	1.1 (0.2)	1.0 (0.2)	1.0 (0.2)	1.0 (0.3)	0.34
	CREB	1.0 (0.3)	1.1 (0.3)	1.1 (0.5)	1.0 (0.4)	0.52
	IFNγ	0.9 (1.3)	0.4 (0.2)	0.8 (1.0)	0.9 (1.0)	0.16
	IL-1β	1.0 (0.6)	0.5 (0.4)	0.8 (0.8)	0.4 (0.5)	0.02 [a]
	TNFα	0.9 (0.4)	1.0 (0.5)	1.0 (0.5)	0.7 (0.9)	0.18

Table S3 : Comparison of brain markers using the Kruskal-Wallis test. Results are expressed as median of 2eΔΔCt (interquartile range), n = 11-14. [a] Despite the Kruskal-Wallis significance, the Dunn post hoc test did not reveal any significant difference between groups.

Gut regions	Marker	NS-PBS	S-PBS	NS-Probio	S-Probio	Kruskal-Wallis test P value
Ileum	IL-1β	0.9 (1.2)	1.0 (0.6)	1.2 (0.7)	1.0 (1.2)	0.81
	TNFα	0.8 (0.8)	0.8 (0.6)	1.1 (0.9)	0,5 (0.8)	0.23
	IFNγ	0.8 (1.3)	0.9 (1.2)	0.6 (0.6)	0.8 (0.6)	0.73
	Claudin-2	1.0 (0.2)	0.9 (0.4)	0.9 (0.6)	0.9 (0.3)	0.78
	0ccludin	1.0 (0.6)	1.3 (0.5)	1.2 (0.6)	1.3 (0.8)	0.65
	ZO-1	1.3 (0.3)	1.0 (0.1)	1.0 (0.3)	1.1 (0.3)	0.13
	MLCK	1.0 (0.6)	0.8 (0.9)	1.0 (0.7)	1.0 (1.1)	0.97
Colon	IL-1β	1.0 (0.6)	0.9 (0.4)	1.2 (0.8)	0.8 (0.5)	0.19
	TNFα	0.9 (0.7)	0.8 (0.6)	1.3 (1.3)	0.8 (0.5)	0.26
	IFNγ	1.0 (0.4)	1.1 (0.4)	1.5 (1.4)	1.0 (0.5)	0.18
	Claudin-2	0.9 (0.6)	0.9 (0.4)	1.0 (0.6)	1.3 (0.7)	0.066
	0ccludin	1.0 (0.5)	0.9 (0.5)	0.9 (0.6)	1.1 (0.5)	0.51
	ZO-1	1.0 (0.2)	1.0 (0.3)	0.9 (0.3)	1.1 (0.2)	0.15
	MLCK	1.0 (0.6)	0.6 (0.6)	0.6 (0.3)	0.5 (0.7)	0.17

Table S4 : Comparison of inflammation and gut permeability using the Kruskal-Wallis test. Results are expressed as median of 2eΔΔCt (interquartile range), n = 12-14.

Taxa level	S-PBS vs NS-PBS Taxa	p-value	S-Probio vs NS-Probio Taxa	p-value	S-Probio vs S-PBS Taxa	p-value
Family	Verrucomicrobiaceae	p= 0.007	**Bacteroidaceae**	p= 0.0001 *	Rikenellaceae	p= 0.0072
	**Bacteroidaceae**	p= 0.014	Bdellovibrionaceae	p= 0.0123	**Staphylococcaceae**	p= 0.0085
			Desulfovibrionaceae	p= 0.258		
			**Staphylococcaceae**	p= 0.0313		
Genus	*Akkermansia*	p= 0.007	***Bacteroides***	p= 0.0001 *	*Ruminococcus*	p= 0.0203
	Allobaculum	p= 0.0079	***Ruminococcus2***	p= 0.0082	*Alistipes*	p= 0.0072
	Bacteroides	p= 0.014	***Coprobacter***	p= 0.0063	***Staphylococcus***	p= 0.0085
	Olsenella	p= 0.0194	*Olsenella*	p= 0.0096		
	Gemmiger	p= 0.0258	***Catenibacterium***	p= 0.0104		
	Butyrivibrio	p= 0.0291	*Vampirovibrio*	p= 0.0123		
	Coprococcus	p= 0.0326	*Bilophila*	p= 0.0203		
			Anaerosporobacter	p= 0.0228		
			Macellibacteroides	p= 0.0256		
			Clostridium IV	p= 0.0291		
			Staphylococcus	p= 0.0313		
			Coprococcus	p= 0.0366		
			Murimonas	p= 0.0366		
			Sporobacter	p= 0.0456		

Table S5 : Relative abundance significantly regulated between treatments at week 6. * indicates a (FDR-adjusted p-value) q-value <0.01, bold indicates a higher relative abundance in the first group

	Marker	NS-PBS	S-PBS	NS-Probio	S-Probio	Kruskal-Wallis test P value
Faeces	Total SCFAs (µmol/g caecal content)	11.4 (4.7)	8.5 (5.4)	12.9 (9.6)	6.9 (5.2)	0.019
	% Acetate	74.9 (3.7)	80.0 (8.9)	75.1 (6.0)	81.3 (16.4)	0.17
	% Propionate	13.0 (6.3)	10.1 (4.2)	10.3 (6.3)	10.8 (5.9)	0.24
	% Butyrate	8.4 (4.8)	8.0 (5.3)	12.4 (6.2)	8.4 (10.5)	0.20
	% Iso-SCFAs	1.1 (1.2)	0.9 (0.8)	1.2 (1.0)	0.7 (0.5)	0.49
Caecum content	Total SCFAs (µmol/g caecal content)	42.3 (20.7)	32.7 (19.4)	32.7 (10.8)	30.6 (10.0)	0.28
	% Acetate	65.7 (2.7)	60.9 (4.8)	63.6 (4.9)	60.9 (4.7)	0.014
	% Propionate	10,68 (2.2)	11,73 (3.8)	13,72 (3.7)	12,49 (4.6)	0.044
	% Butyrate	0.7 (0.2)	0.8 (0.4)	0.9 (0.3)	0.8 (0.5)	0.21
	% Iso-SCFAs	1.3 (0.4)	1.4 (0.8)	1.7 (0.6)	1.5 (1.1)	0.16

Table S6 : Comparison of total SCFAS and percentages of acetate, propionate, butyrate and iso-SCFAs using the Kruskal-Wallis test. Results are expressed as median (interquartile range), n = 13-14.
