# Supplementary figures and images for: Effects of a Lacticaseibacillus Mix on Behavioural, Biochemical, and Gut Microbial Outcomes of Male Mice following Chronic Restraint Stress
Source: Nutrients. 2023 Oct 31;15(21):4635. doi: 10.3390/nu15214635 (PMC10648220; doi:10.3390/nu15214635)

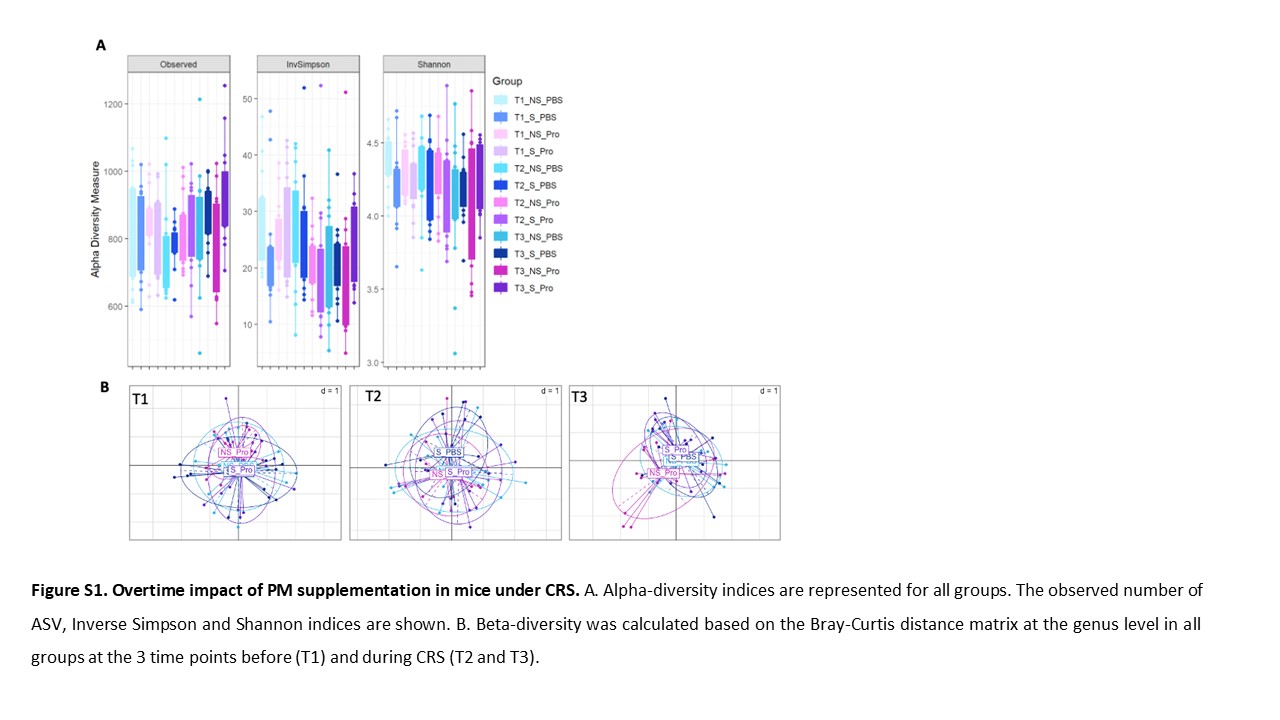

Supplement: Supplementary file 1 [file nutrients-15-04635-s001.zip › Supplementary Figure S1.jpg]
